# Positive Selection at Key Residues in the HIV Envelope Distinguishes Broad and Strain-Specific Plasma Neutralizing Antibodies

**DOI:** 10.1128/JVI.01685-18

**Published:** 2019-03-05

**Authors:** Batsirai M. Mabvakure, Cathrine Scheepers, Nigel Garrett, Salim Abdool Karim, Carolyn Williamson, Lynn Morris, Penny L. Moore

**Affiliations:** a Center for HIV and STIs, National Institute for Communicable Diseases, National Health Laboratory Service, Johannesburg, South Africa; b School of Pathology, Faculty of Health Sciences, University of the Witwatersrand, Johannesburg, South Africa; c Centre for the AIDS Programme of Research in South Africa (CAPRISA), KwaZulu Natal, South Africa; d Department of Epidemiology, Columbia University, New York, New York, USA; e Division of Medical Virology, Institute of Infectious Disease and Molecular Medicine, University of Cape Town, Cape Town, South Africa; Emory University

**Keywords:** broadly neutralizing antibodies, envelope glycoprotein, epitope diversity, HIV vaccine, HIV-1 intrapatient evolution, sequential vaccination

## Abstract

The development of HIV broadly neutralizing antibodies (bNAbs) has previously been shown to be associated with viral evolution and high levels of genetic diversity in the HIV envelope (Env) glycoprotein. However, few studies have examined Env evolution in those who fail to develop neutralization breadth in order to assess whether bNAbs result from distinct evolutionary pathways. We compared Env evolution in eight HIV-1-infected participants who developed bNAbs to six donors with similar viral loads who did not develop bNAbs over three years of infection. We focused on Env V1V2 and C3V4, as these are major targets for both strain-specific neutralizing antibodies (nAbs) and bNAbs. Overall evolutionary rates (ranging from 9.92 × 10^−3^ to 4.1 × 10^−2^ substitutions/site/year) and viral diversity (from 1.1% to 6.5%) across Env, and within targeted epitopes, did not distinguish bNAb donors from non-bNAb donors. However, bNAb participants had more positively selected residues within epitopes than those without bNAbs, and several of these were common among bNAb donors. A comparison of the kinetics of strain-specific nAbs and bNAbs indicated that selection pressure at these residues increased with the onset of breadth. These data suggest that highly targeted viral evolution rather than overall envelope diversity is associated with neutralization breadth. The association of shared positively selected sites with the onset of breadth highlights the importance of diversity at specific positions in these epitopes for bNAb development, with implications for the development of sequential and cocktail immunization strategies.

**IMPORTANCE** Millions of people are still being infected with HIV decades after the first recognition of the virus. Currently, no vaccine is able to elicit bNAbs that will prevent infection by global HIV strains. Several studies have implicated HIV Env diversity in the development of breadth. However, Env evolution in individuals who fail to develop breadth despite mounting potent strain-specific neutralizing responses has not been well defined. Using longitudinal neutralization, epitope mapping, and sequence data from 14 participants, we found that overall measures of viral diversity were similar in all donors. However, the number of positively selected sites within Env epitopes was higher in bNAb participants than in strain-specific donors. We further identified common sites that were positively selected as bNAbs developed. These data indicate that while viral diversity is required for breadth, this should be highly targeted to specific residues to shape the elicitation of bNAbs by vaccination.

## Abstract (precis)

Millions of people are still being infected with HIV decades after the first recognition of the virus. Currently, no vaccine is able to elicit bNAbs that will prevent infection by global HIV strains. Several studies have implicated HIV Env diversity in the development of breadth. However, Env evolution in individuals who fail to develop breadth despite mounting potent strain-specific neutralizing responses has not been well defined. Using longitudinal neutralization, epitope mapping, and sequence data from 14 participants, we found that overall measures of viral diversity were similar in all donors. However, the number of positively selected sites within Env epitopes was higher in bNAb participants than in strain-specific donors. We further identified common sites that were positively selected as bNAbs developed. These data indicate that while viral diversity is required for breadth, this should be highly targeted to specific residues to shape the elicitation of bNAbs by vaccination.

## INTRODUCTION

A preventative HIV vaccine is likely to require broadly neutralizing antibodies (bNAbs) that have the ability to neutralize the majority of HIV strains (1, 2). Animal studies have shown that bNAbs are able to protect from virus infection (3); however, no vaccine has thus far been able to elicit bNAbs. In contrast, longitudinal studies have shown that bNAbs develop in approximately 10 to 30% of chronically HIV-infected patients (4–6). These bNAbs target five sites on the HIV trimer, namely, the V1V2 region, the CD4 binding site, the membrane proximal external region (MPER), the V3 glycan supersite, and the gp41-gp120 interface, which includes the fusion peptide (7). Although most donors develop some degree of cross-reactivity over many years of infection (8), some donors have neutralizing antibody responses that remain strain-specific, neutralizing only the infecting strain, despite equivalent duration of infection and high viral loads (9). The reason strain-specific responses fail to mature into bNAbs in all HIV-infected individuals is an important unanswered question in the field.

Large cohort studies have shown that neutralization breadth is associated with several host factors, including ethnicity, HLA genotype, CD4 T cell loss, and circulating T follicular helper cells (4–6, 10–13). However, viral factors, such as subtype, high viral loads, virus diversity, and duration of HIV-1 infection, have been shown to be strongly associated with breadth, highlighting the importance of high levels of antigenic stimulation in driving the maturation of bNAbs (5, 6, 14, 15).

Studies of the developmental pathways of bNAbs have shown that these develop from strain-specific precursors that mature to acquire breadth through a prolonged coevolutionary pathway, with emerging viral mutations driving antibodies to tolerate epitope diversity (2, 16–24). This has led to a vaccine framework that aims to incorporate variation within immunogens, an approach that has recently shown promise in small-animal immunization studies (25–30). However, similarly detailed studies of viral evolution in donors who maintain strain-specific responses, especially in the context of high levels of antigenic stimulation, have not been performed. Comparative analyses of virus evolutionary pathways in these individuals and those who develop bNAbs may provide useful insights into the viral characteristics and mechanisms that drive antibody maturation toward breadth.

Robust strain-specific neutralizing responses develop in almost all infected individuals, regardless of whether they later develop breadth, and exert significant selection pressure on the HIV envelope glycoprotein (Env) (31, 32). This results in increased Env diversity and divergence from the transmitted/founder (T/F) virus and selection of neutralization-resistant strains. Escape is associated with increased variable loop length and glycosylation that may shield underlying epitopes, making it difficult to neutralize the virus (33–38). Importantly, many of the strain-specific neutralizing responses target the same overall regions of the HIV envelope as bNAbs, such as the V1V2 and C3V4 regions (24, 39), and drive viral diversification within these regions (24, 38, 40).

To assess why these strain-specific responses fail to acquire breadth, we have used longitudinal single-genome-derived full Env sequencing data and matched antibody kinetics and mapping data from 14 participants in the Centre for the AIDS Programme of Research in South Africa (CAPRISA) 002 cohort, six of whom developed strain-specific antibodies and eight who developed bNAbs. Interestingly, we did not observe significant differences in nucleotide substitution rates in participants with or without bNAbs, and we observed high viral diversity across Env and within epitopes in both groups. However, we identified several sites under positive selection that were common to donors with bNAbs. The tight clustering of these sites on the Env trimer suggests that the precise targeting of viral epitopes by early bNAb precursors, and the subsequent viral escape pathways, determines whether these antibodies acquire breadth. Defining these sites highlights developmental differences in strain-specific antibodies and bNAbs which may inform future vaccine design strategies.

## RESULTS

### Env diversity and evolutionary rates do not distinguish broad and strain-specific donors with equivalent viral loads.

**FIG 1:**
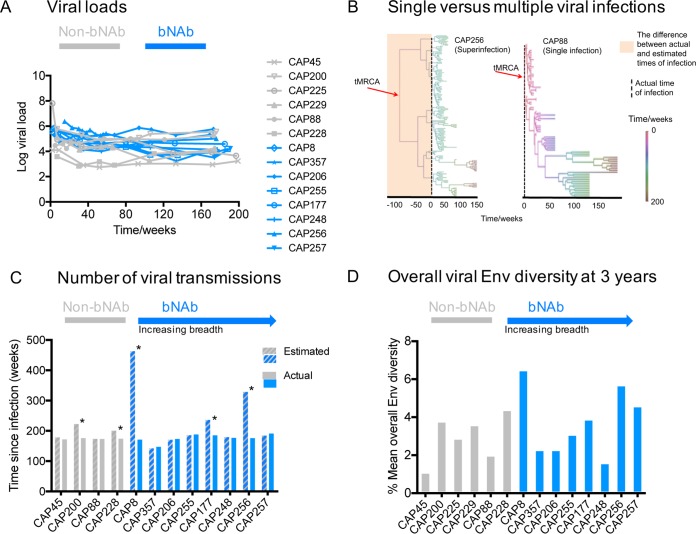
Viral diversity and evolutionary rates are not the sole determinants of bNAb development. (A) Longitudinal viral loads of all participants, with bNAb donors in blue and strain-specific donors in gray. (B) Maximum clade credibility trees of CAP88 and CAP256 constructed in BEAST showing the estimated time of infection (tMRCA) compared to actual time of infection. Similar analyses were performed for all donors. (C) Comparison of estimated and actual time of infection in 12 participants. The asterisk (*) denotes possible dual infection. (D) Comparison of overall amino acid envelope diversity at 3 years in participants with/without bNAbs.

We first assessed viral diversity in 6 non-bNAb donors (with <10% breadth) and 8 bNAb donors (with >40% breadth) over 3 years of infection (Table 1). Since viral diversity is closely linked to viral load, we selected, where possible, non-bNAb participants with similarly high viral loads to the bNAb donors. Two participants, CAP45 and CAP228, who had consistently low viral loads, were also included, as we have extensively characterized their neutralizing responses (Fig. 1A). We generated between 29 and 171 single-genome-derived *env* sequences per donor from multiple time points spanning 3 years (Table 1). Maximum likelihood analysis of transmitted/founder viruses from bNAb and non-bNAb donors confirmed that these were phylogenetically unlinked (data not shown).

**TABLE 1:** Description of HIV-infected participants, plasma neutralization breadth, set point viral load, and antibody targets

Participant	% neutralization breadth at 3 yr^*a*^	No. of *env* sequences	No. of time points	Set point viral load at 12 mo postinfection (RNA copies/ml)	Antibody target(s)^*b*^	Source or reference
CAP257	82	63	6	8,260	**V1V2, CD4 binding site**	24
CAP256	77	171	13	178,000	**V1V2**	16, 46
CAP248	59	120	18	64,600	**gp120-gp41 interface**	43
CAP177	52	144	7	18,000	**C3V4,** V1V2	23, 38
CAP255	47	148	13	18,200	**C3V4**	50
CAP206	47	130	9	315,000	**MPER,** C3V4	4; J. N. Bhiman, unpublished data
CAP357	45	76	7	11,596	Not mapped	M. Madzhivandila, unpublished data
CAP8	42	70	5	39,300	Not mapped	50
CAP88	8	83	8	38,700	V1V2, C3V4	38
CAP228	7	100	6	1,520	V1V2, C3V4	J. N. Bhiman, unpublished data
CAP229	7	35	3	24,600	V1V2	J. N. Bhiman, unpublished data
CAP225	5	29	3	21,500	Not mapped	J. N. Bhiman, unpublished data
CAP200	2	53	4	108,000	C3V4	J. N. Bhiman, unpublished data
CAP45	0	41	6	556	Not mapped	38

a Reference 4.

b bNAbs are shown in bold.

Since dual infection results in increased genetic diversity, we used BEAST to compare the estimated time of infection (tMRCA), based on the sequence diversity, to the actual time of infection for each donor (41, 42). For dual infection, sequence analysis predicts a greater estimated duration of infection than the known time of infection; this is indicated by orange shading for CAP256 (a donor known to be superinfected), but not for CAP88, in Fig. 1B. Twelve out of 14 data sets had sufficiently strong temporal signals (as determined by the positive slope [rate] and correlation coefficient) for phylogenetic molecular clock analysis. The two donors that were excluded from this analysis were CAP225 and CAP229. BEAST analysis indicated single infections in 6 donors, CAP88, CAP206, CAP248, CAP255, CAP357, and CAP257 (Fig. 1C, bNAb participants are ranked by breadth), consistent with the fact that most HIV-1 infections are initiated by a single variant (44, 45). In contrast, we saw evidence of dual infection (indicated by asterisks in Fig. 1C) for 5 donors, CAP200, CAP228, CAP8, CAP177, and CAP256. Dual infections were observed both in individuals with bNAbs (CAP8, CAP177, and CAP256) and without bNAbs (CAP200 and CAP228).

We next compared the levels of overall Env glycoprotein diversity in participants with and without bNAbs at 3 years postinfection (Fig. 1D). We observed the highest overall diversity in CAP228, CAP8, CAP256, and CAP257, with a mean overall diversity >4% amino acid differences per site at 3 years postinfection (Fig. 1D). With the exception of CAP257, this is likely a result of their infection with multiple viruses or variants (Fig. 1C). Among these donors, CAP228, who failed to develop breadth, had relatively high diversity despite low viral loads (Fig. 1A). However, overall, there was no statistically significant difference in Env diversity between participants with or without bNAbs (*P* = 0.7758, two-tailed Mann-Whitney test). Similarly, evolutionary rates across the envelope glycoprotein, based on nucleotide substitution rates estimated in BEAST, showed no significant differences between these groups (data not shown).

### Comparison of Env evolution within antibody-targeted regions between bNAb and non-bNAb donors.

**FIG 2:**
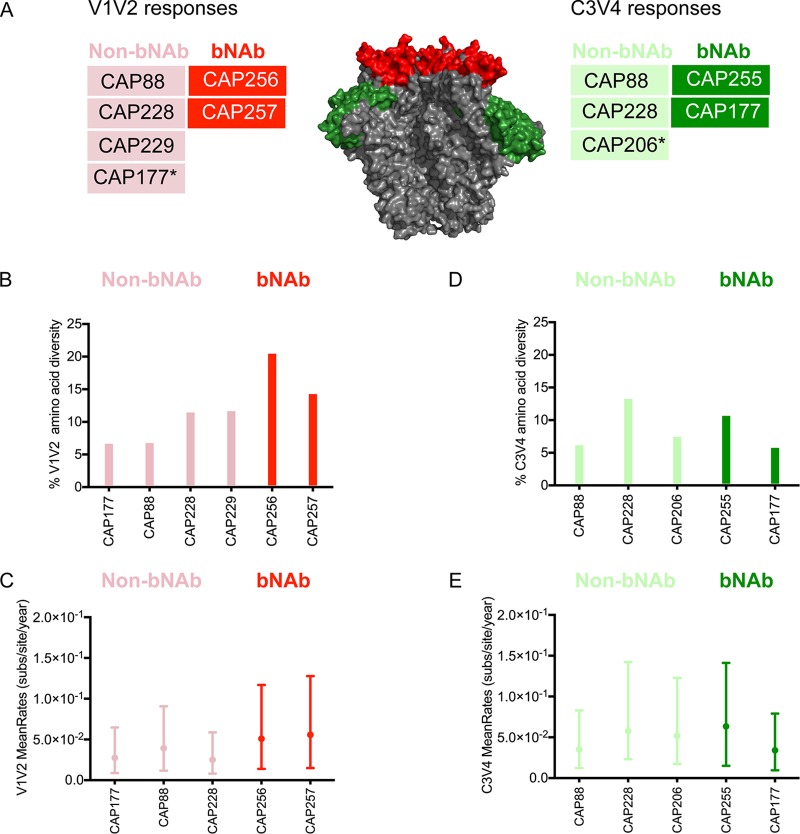
Comparison of diversity and nucleotide substitution rates in epitope regions targeted by participants with/without bNAbs. (A) Schematic of an Env trimer showing the antibody targets defined in previous studies. Participants with V1V2 responses are shown in red, and those with C3V4 responses are shown in green. The dark shading and light shading represent participants with and without bNAbs, respectively. The asterisk (*) denotes participants who have bNAb responses to other regions but also developed strain-specific antibodies to V1V2 or C3V4. Epitope diversity (average number of amino acid differences per site) and nucleotide substitution rates within V1V2 (B and C) and C3V4 (D and E) in participants with/without bNAbs. Broad participants arranged in order of increasing neutralization breadth. subs, substitutions.

We have previously mapped the targets of both strain-specific and bNAb responses in 8 participants to the V1V2 and/or C3V4 regions (Table 1 and Fig. 2A). This enabled us to compare the Env evolutionary pathways between bNAb and non-bNAb donors specifically within these targeted regions. The V1V2 and C3V4 regions were defined according to the HXB2 reference sequence as amino acid positions 131 to 196 and 329 to 418, respectively. We first compared two donors with V1V2-directed bNAbs (CAP256 and CAP257) with four donors who had strain-specific V1V2 responses (CAP88, CAP228, CAP229, and CAP177) (4, 23, 24, 46) (Table 1). Although CAP177 developed bNAbs to the C3V4 region, this participant also had a strain-specific V1V2 response (38) and is included as a strain-specific donor for this epitope. The two participants with V1V2-directed bNAbs (CAP256 and CAP257) had higher levels of V1V2 amino acid diversity (20.7% and 14.5%, respectively) than did non-bNAb participants (ranging from 6.9 to 11.9% amino acid differences per site, though this difference failed to reach significance; Fig. 2B). Similarly, the two bNAb participants showed slightly higher V1V2 evolutionary rates than those of non-bNAb donors; however, these differences were also not significant, with estimates within the same high posterior density intervals (Fig. 2C).

We similarly compared two donors who developed N332-specific bNAbs (CAP177 and CAP255; dark green in Fig. 2A) with three donors shown to have strain-specific C3V4 responses (CAP88, CAP206, and CAP228; light green in Fig. 2A). CAP206 developed bNAbs that targeted the MPER (4), but in this analysis, we focused on the strain-specific C3V4 response. bNAb donors showed no difference in overall C3V4 diversity and nucleotide substitution rates from those of the strain-specific C3V4 responders (Fig. 2D and E). Overall, analysis of viral evolution within antibody-targeted regions did not show evidence of unique viral evolutionary pathways for donors with N332-directed bNAbs.

### Participants with bNAbs show high levels of positive selection within targeted epitopes.

We compared levels of positive selection within epitope regions in bNAb and non-bNAb donors by estimating the ratio of nonsynonymous to synonymous substitutions per site (47). We first compared two approaches for assessing selection, cumulative and a snapshot selection analyses. In the cumulative analysis, all sequences at preceding time points were combined, whereas in the snapshot analysis, we analyzed sequence data only from two consecutive time points. The cumulative analysis identified the same positively selected sites as the snapshot analysis, as well as additional sites, and unlike the snapshot analysis, it retained all sequences for analysis (data not shown). All subsequent analyses were therefore done using the cumulative analysis.

**FIG 3:**
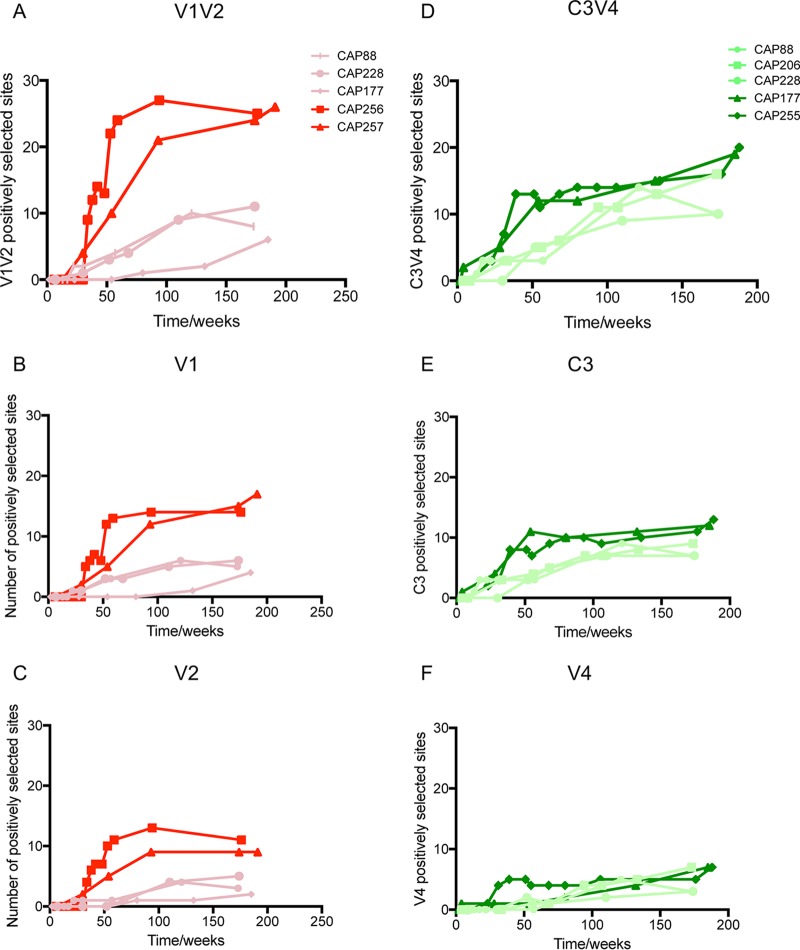
bNAb participants have more sites under positive selection than non-bNAb individuals. Positive selection was higher in the V1V2 region of participants who developed bNAbs (dark red) than in those with strain-specific neutralizing antibodies (dull red) (A to C). In participants that had C3V4 responses, positive selection was higher in the C3V4 region of bNAb participants (dark green) than in those with strain-specific neutralizing antibodies (dull green) only in the first year of infection (D to F).

As expected, the number of positively selected sites increased over time in all participants regardless of whether they developed breadth or not (Fig. 3A and D). However, participants with V1V2-directed bNAbs exhibited a rapid increase in the number of positively selected sites (peaking at 26 to 27 amino acid residues) in V1V2 within the first year, compared to those without bNAbs (6 to 11 amino acid residues), and this increased level of positive selection persisted over 3 years of infection (Fig. 3A to C). Similarly, participants with C3V4-directed bNAb responses had an increased number of positively selected sites (peaking at 11 to 12 amino acid residues at 1 year) in C3V4 during the first year of infection compared to that of strain-specific donors (3 to 4 amino acid residues at 1 year) (Fig. 3D to F), though this difference was less pronounced than that observed in V1V2 and was not sustained in later years. These differences between bNAb and non-bNAb donors were driven largely by the C3 region (Fig. 3D to F), consistent with the fact that bNAbs to this region are known to be focused on the N332 glycan in C3, whereas strain-specific antibodies also target the more sequence-variable V4 region (39, 48).

### Common sites in the V2, C3, and V3 regions are targeted by bNAbs.

We next compared the specific sites under positive selection between participants with and without bNAbs. We focused on the V2 and C3 regions only, as the V1 and V4 regions were highly variable and prone to indels, making accurate identification of specific sites difficult. Using FUBAR, we identified positively selected sites in the V2 and C3 regions in each of the donors and compared their positions between bNAb and non-bNAb donors.

**FIG 4:**
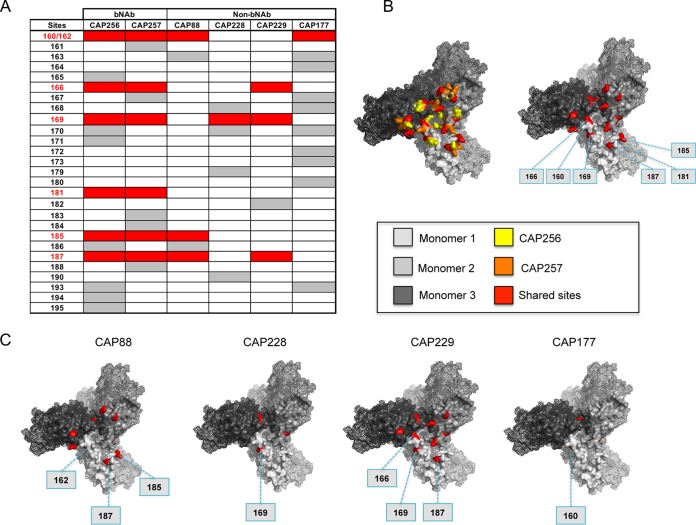
Comparison of positively selected sites in the V2 region of participants with and without bNAbs. (A) Table showing all sites under positive selection in the V2 region of participants with V1V2 responses. Positively selected sites common to both bNAb donors are highlighted in red. (B) Trimer model showing all sites under positive selection in participants with V2 responses (left) and common sites under positive selection in CAP256 and CAP257 (right). (C) bNAb sites also under positive selection in non-bNAb participants.

In the V2 region, we identified 28 positively selected sites across all donors with V1V2 responses. bNAb donors CAP256 and CAP257 had 13 and 11 positively selected sites in V2, respectively (Fig. 4A). Of these, six sites (160 and 162, which together form the highly conserved N160 glycosylation sequon, 166, 169, 181, 185, and 187) were common in both V2 bNAb participants (CAP256 and CAP257) (highlighted in red in Fig. 4A and B). Four of these sites were also targeted by at least one of the non-bNAb donors, indicating that strain-specific V1V2 antibodies overlapped somewhat with bNAbs. Many of these sites (160/162, 166, 169, and 181) have previously been reported to mediate escape from V1V2-directed bNAbs (17, 49–51), and all are located proximal to one another at the apex of the envelope trimer (Fig. 4B). However, overall positive selection at these sites, particularly at sites 166 and 181, was less common in donors who developed strain-specific V1V2 responses (Fig. 4C).

**FIG 5:**
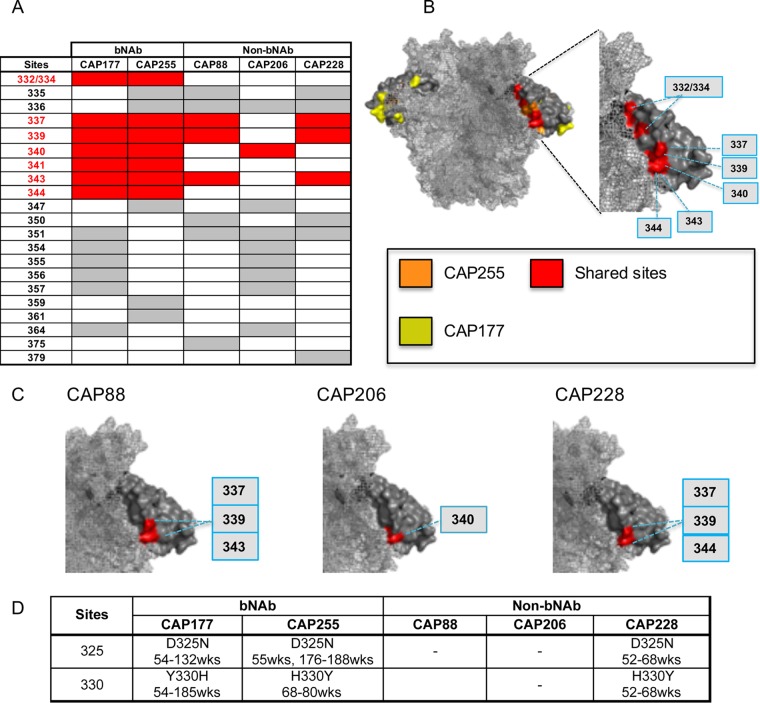
Comparison of positively selected sites in the C3 region of participants with and without breadth. (A) Table showing all sites under positive selection in the C3 region of participants with C3V4 responses. Positively selected sites common to both bNAb donors are highlighted in red. (B) Trimer model showing all sites under positive selection in participants with C3V4 responses (left) and common sites under positive selection in bNAb donors CAP256 and CAP257 (right). (C) bNAb sites also under positive selection in non-bNAb participants. (D) Analysis of sites that toggle in the V3 GDIRQAH motif of participants with C3V4-directed responses. wks, weeks.

Similarly, 22 positively selected sites were identified within the C3 region of the 5 participants with C3V4 responses (Fig. 5A). Of these, seven sites (332/334, which form part of mutually exclusive glycosylation sequons, 337, 339, 340, 341, 343, and 344) were common in both participants who developed bNAbs targeting the N332 supersite (highlighted in red in Fig. 5A and B). Unlike the two bNAb participants, donors with strain-specific responses to the C3V4 region did not show evidence of positive selection at residue 332 or 334. In addition to the glycan, residues 341 and 344 were not under positive selection in any of the three strain-specific donors (CAP88, CAP206, and CAP228). Sites 337, 339, 340, and 343, under positive selection in both bNAb donors, were also sometimes under positive selection in participants who failed to develop bNAbs (Fig. 5C). Of note, we identified other common sites (351, 354, 355, 356, and 357) under selection in both CAP177 and CAP206 that clustered together within the C3 region on the envelope trimer (highlighted in yellow in Fig. 5B), suggesting another distinct epitope within this region.

bNAbs to the N332 supersite have also been shown to depend on the ^324^GDIRQAH^330^ motif at the base of the V3 loop (52). We therefore analyzed selection at these sites. Although none reached statistical significance for positive selection, we observed toggling at sites 325 and 330 in both bNAb donors (CAP177 and CAP255) but also in strain-specific donor CAP228. No such toggling was observed in CAP206 and CAP88 (Fig. 5D). Sites 325 and 330 have been reported to impact neutralization sensitivity when these mutations are accompanied by changes at other sites, such as 301, 324, 326, and 327 (52). The similarity of toggling of V3 residues in CAP177, CAP255, and CAP228 might suggest a similar epitope for both broad antibodies and some strain-specific antibodies, raising the question of how they differ in their mode of recognition.

### Increased positive selection is associated with neutralization breadth.

**FIG 6:**
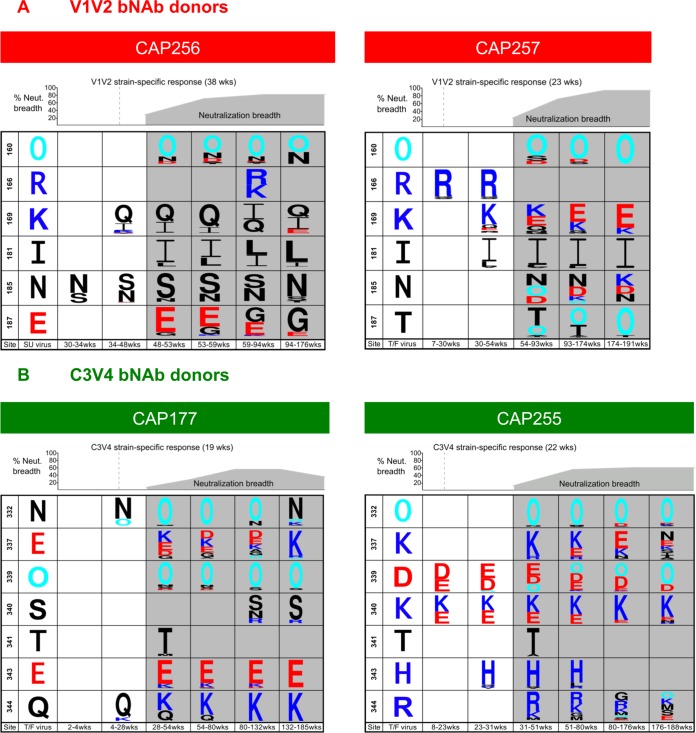
Increased positive selection at the onset of neutralization (Neut.) breadth. Breadth is shown in gray, glycans are shown in cyan, positively charged amino acids are shown in blue, negatively charged amino acids are shown in red, and noncharged amino acids are shown in black. (A) Participants with V1V2-directed bNAbs showed increased numbers of positively selected bNAb sites (160, 166, 169, 181, 185, and 187) as breadth developed. (B) Participants with C3V4 bNAbs accumulated pressure at sites 332, 337, 339, 340, 341, 343, and 344 with breadth.

We next assessed whether positive selection at key sites increased with the acquisition of breadth. For this analysis, we used previously published data defining the kinetics of bNAbs (gray shading, Fig. 6) and strain-specific responses to the same epitope (dotted line, Fig. 6) (4, 16, 17, 23, 24, 33, 38). Selected residues are shown compared to the T/F virus, except for CAP256, where the comparison is with the superinfecting virus previously shown to elicit bNAbs in this donor (16). For V1V2 bNAb donors CAP256 and CAP257, we observed positive selection at sites 169/185 and 166/169/181, respectively, prior to the development of breadth, likely driven by the earlier strain-specific responses (Fig. 6A). In CAP256, the positively charged K169 in the superinfecting virus was replaced with a 169Q, which is rare among subtype C viruses but present in the primary infecting virus and confers partial escape from V2 directed antibodies (16). Similarly, in CAP257, a charge-changing minority K169E mutation emerged prior to the development of breadth. In both donors, positive selection expanded to additional sites, including the N160 glycan, with the onset of neutralization breadth. In CAP257, additional mutually exclusive glycans at positions at 185 and 187 were selected for after bNAb development.

In C3V4 donors CAP177 and CAP255, strain-specific responses drove positive selection at two to three sites (N332 and 344 for CAP177 and sites N339 and 340 for CAP255) (Fig. 6B). However, the emergence of bNAbs was associated with a significant increase in levels of positive selection. For CAP177, we have previously shown that the T/F does not contain a N332 glycan, and that early strain-specific neutralizing antibodies (nAbs) drive selection of this glycan, which is subsequently lost as bNAbs develop (23). For CAP255, the N332 glycan is under positive selection but is nonetheless largely maintained in most late viral variants. Overall, we observed a significant increase in the number of key sites exhibiting selection pressure with the onset of breadth (Fig. 6), both within the V1V2 and C3V4 bNAb donors.

## DISCUSSION

The study of virus-antibody coevolution in infected donors who develop bNAbs has provided crucial insights into how viral variation drives antibody lineages toward breadth. However, most studies have focused on bNAb donors, making the assumption that specific Env evolutionary patterns are unique to this group. The existence of infected donors who develop strain-specific responses raises the question of whether viral evolutionary pathways in these donors differ from those of the well-described bNAb donors. The purpose of this study was therefore to compare the evolutionary pathways of HIV envelope glycoproteins in bNAb and non-bNAb donors to identify whether there are distinct viral features that are associated with breadth. Moreover, we used previously generated data describing the targets of both strain-specific antibodies and bNAbs to compare viral evolution specifically within epitope regions. We showed that neither overall viral diversity nor local diversity in targeted regions is sufficient for breadth. However, we observed higher numbers of positively selected sites in broad neutralizers and identified several positively selected sites in the V2 and C3 regions that were common to bNAb donors and limited or absent in participants without neutralization breadth. Selection pressure at these sites also increased with onset of neutralization breadth, highlighting the role of targeted viral selection in the development of breadth.

One of the consistent factors associated with the development of bNAbs in cohort studies is the presence of high levels of viral diversity (5, 6, 9, 14, 15). However, in this study, Env diversity at 3 years of infection showed no significant differences between bNAb and non-bNAb donors. While this may appear to contradict findings from previous cohort studies, it is important to note that most non-bNAb donors in this study were selected to have a viral load similar to that of the bNAb donors. As viral load and diversity are linked, this analysis indicates that in donors with similar viral loads, overall diversity does not distinguish bNAb donors from non-bNAb donors, and it suggests that additional factors influence breadth. The lack of difference in overall Env diversity between the two groups is consistent with several studies showing that bNAbs often represent only a small proportion of the overall neutralizing responses in an infected donor (53; see also D. Kitchin and P. L. Moore, unpublished data). Although most of the strain-specific neutralizing antibodies that comprise the overall neutralizing response fail to acquire breadth (as is evident from the low proportion of bNAb donors in infected cohorts), they nonetheless drive substantial diversity through viral escape. Indeed, there appears to be no clear association between overall autologous neutralization titers and breadth (4, 54, 55).

Superinfection has been reported to be important in the development of breadth, although the effect is small (16, 56, 57). Our study included two donors where the tMRCA and actual time of infection differed significantly, suggesting (CAP8) or confirming (CAP256) superinfection. Both donors developed bNAbs, and CAP256 has been intensively studied over many years (16, 17, 46, 58). However, in CAP256, we have recently demonstrated that superinfection played no significant role in driving breadth (55). Furthermore, within the CAPRISA cohorts, we see no association between superinfection and breadth (55).

Many studies defining viral escape from bNAbs have confirmed that despite the conserved nature of these epitopes, HIV can utilize multiple pathways to escape (23, 24, 59). For this reason, bNAbs fail to confer any clinical benefit to those donors in whom they develop (4). In donors who develop breadth, extensive toggling with epitopes has been shown to precede the development of breadth (17, 18). Our observation of higher numbers of positively selected sites in bNAb donors, particularly within the first year of infection, supports the notion that this early but targeted viral toggling is required for the development of breadth, and it differentiates bNAb donors from those who remain strain specific.

The underlying reason for a higher number of positively selected sites in bNAb donors is of interest for HIV vaccine design. This is unlikely to be a consequence of higher titers, as many of the strain-specific responses we have previously mapped have equivalently high titers to bNAb responses (38, 60). The targeting of intrinsically more conserved epitopes may result in fitness costs for some escape mutations and, therefore, higher levels of compensatory mutations at proximal sites (61). Alternatively, it is possible that antibodies with the potential to mature toward breadth bind the virus using a wider “footprint,” i.e., they have more contact sites and therefore drive early escape mutations at more sites. The finding that mutations at many of these positively selected sites, when introduced into heterologous viruses, confer neutralization escape from bNAbs may support the latter possibility (17, 18, 49, 52, 62–64).

The identification of sites common to bNAb donors and their clustered location on the trimeric Env may emphasize the importance of accurate early targeting by bNAbs. Studies of the ontogeny of V1V2-directed bNAb donors have confirmed the need for precursors encoding long third complementarity-determining regions to penetrate the glycans shielding the V2 C-strand (where many of the positively selected sites we identify here are located). The tight clustering of positively selected sites for C3V4 bNAb donors in this study may suggest that initial targeting is important here, too, though mature members of V3-directed bNAb lineages show much more promiscuity in their angles of approach as they mature to accommodate sequence variation and glycosylation (65–71).

We selected 14 participants for the in-depth epitope study because of the availability of both sequence and mapping data. Furthermore, both V1V2- and N332-directed bNAbs are among the most common bNAb specificities elicited during infection (4, 6, 54, 72, 73) and may require relatively less somatic hypermutation to acquire breadth (16, 20, 74). This makes these bNAb specificities attractive vaccine targets. However, this study could be improved by performing similar analyses on additional participants with/without bNAbs, including other less commonly elicited bNAb specificities. Since the number of participants in this study was relatively small, larger cohorts and inclusion of donors infected by other viral subtypes would be valuable in the future. We also utilized single-genome Sanger sequencing data with limited depth, whereas sequences generated through next-generation sequencing would give a more accurate representation of viral diversity. Nonetheless, this study has highlighted key differences between the evolutionary pathways of HIV *env* glycoproteins in participants with and without bNAbs. Furthermore, it has clarified that amino acid diversity at key positions within the V1V2 and C3V4 epitopes is more important than overall *env* diversity. The association of shared positively selected sites with the onset of breadth highlights the importance of diversity at these positions in bNAb development. Designing immunogens against HIV that include targeted diversity within these common sites may thus be critical in the maturation of V1V2- and C3V4-directed bNAbs.

## MATERIALS AND METHODS

### HIV-infected participants and ethics.

Longitudinal plasma samples were collected from 14 women chronically infected with HIV-1 subtype C who were enrolled in the CAPRISA 002 and 004 cohorts (75, 76). Of these, 8 participants (CAP8, CAP177, CAP206, CAP357, CAP248, CAP255, CAP256, and CAP257) developed bNAbs (defined by their ability to neutralize more than 40% of a multisubtype panel at 3 years) and 6 donors (CAP45, CAP88, CAP200, CAP225, CAP228, and CAP229) who did not develop bNAbs (with plasma breadth less than 10%) despite approximately 3 years of HIV infection (4) (M. Madzhivandila, unpublished data).

Evidence of HIV infection in participants was determined using two rapid HIV-1 antibody tests (Determine, Abbott 89 Laboratories, Tokyo, Japan; and Capillus, Trinity Biotech, Jamestown, NY). False positives and negatives were eliminated by using PCR for the negative samples (Ampliscreen version 1.5; Roche Diagnostics, Rotkreuz, Switzerland) and an HIV enzyme immunoassay (EIA; BEP2000; Dade Behring, Marburg, Germany) for positive samples. Viral loads were estimated using the Cobas Amplicor HIV-1 Monitor test version 1.5 (Roche Diagnostics). The time of infection was estimated as either the midpoint between the last antibody-negative visit and first antibody-positive visit, or 14 days prior to an RNA-positive antibody-negative sample.

Written informed consent to conduct research on stored specimens was obtained from all women at enrollment, and ethics clearance for the use of plasma samples was obtained from the Human Research Ethics Committee (Medical) from the University of Witwatersrand (MM040202), the University of Cape Town (025/2004), and CAPRISA at the University of KwaZulu-Natal (E013/04).

### Single-genome envelope amplification and sequencing.

HIV-1 RNA was isolated from plasma samples, and full-length envelope genes were amplified from single genomes at multiple time points by nested PCR and sequenced using Sanger sequencing, as described previously (77).

### Sequence alignments and data partitioning.

Sequence alignments were made in MUSCLE (78) and edited manually in BioEdit (79). The longitudinal envelope (*env*) glycoprotein sequences were analyzed as a whole and subsequently partitioned into variable and constant regions for subsequent analyses using the Se-Al software (80). The partitions were defined according to the HXB2 reference sequence as follows: V1, amino acids 131 to 156; V2, amino acids 157 to 196; V3, amino acids 296 to 330; C3, amino acids 329 to 383; and V4, amino acids 385 to 418.

### Estimating diversity, nucleotide substitution rates, and time of infection.

Overall Env, C3V4, and V1V2 diversity was calculated using the Poisson correction distance model implemented in MEGA6 (81, 82). The model assumes equality of substitution rates among sites and equal amino acid frequencies, while correcting for multiple substitutions at the same site (81, 82). The presence of temporal signal was estimated in TempEst using maximum likelihood trees constructed with PhyML (83, 84). Phylogenetic analyses were performed using BEAST version 1.8.4 (85). Best-fit nucleotide substitution models were selected based on the Bayesian information criteria (BIC) and Akaike information criterion, corrected (AICc) values estimated using the MEGA 6 software (81). The best-fit demographic and clock models were selected with marginal likelihood estimations using the generalized path sampling and stepping stone methods with 50 path steps (86, 87). The Monte Carlo Markov chains were run until convergence (effective sample sizes, above 200), and the chain lengths were between 50 and 500 million steps. The log files generated were analyzed in Tracer version 1.5, and the maximum clade credibility (MCC) tree annotated in TreeAnnotator and viewed in FigTree (88).

### Identifying sites under positive selection pressure.

We performed natural selection analyses using Fast Unbiased Bayesian AppRoximation (FUBAR) that identifies codons under positive selection (89, 90). Codons that had more nonsynonymous than synonymous changes and that were supported by a posterior probability greater than 0.9 were reported to be evolving under positive selection. The pipeline for selection analysis involved first analyzing recombination in sequences using the genetic algorithm for recombination detection (GARD) to avoid phylogenetic bias when running FUBAR (89, 90). We employed two strategies in the selection analyses, which we termed the snapshot and cumulative analyses, respectively. The snapshot analysis involved analyzing sequence data from two consecutive time points, whereas the cumulative analysis involved integrating overall sequences in the data set at a particular time point.

### Data availability.

The accession numbers for the *env* sequences are EF203968 to EF203982, FJ443149 to FJ443158, FJ443223 to FJ443239, FJ443275 to FJ443366, FJ443390 to FJ443393, FJ443492, FJ443499, FJ443501 to FJ443504, FJ443508, FJ443519, FJ444632, GQ999980, GK999985, GK999989, HQ625597 to HQ625599, HQ625604, HQ625605, KC863376 to KC863378, KC863380 to KC863392, KC863446 to KC863456, KC863458 to KC863475, KC863542 to KC863553, KC863555 to KC863568, KC863571, KF241776, KF996579, KF996581, KF996584, KF996586 to KF996588, KF996590, KF996591, KF996593 to KF996596, KF996598, KF996600, KF996601, KF996604 to KF996630, KF996632 to KF996699, KF996700 to KF996716, KT698223 to KT698227, KU198436, MF572810 to MF572829, and MK205449 to MK206213.
